# Supplementary material for: Independent control of mean and noise by convolution of gene expression distributions
Source: Nat Commun. 2021 Nov 29;12:6957. doi: 10.1038/s41467-021-27070-5 (PMC8630168; doi:10.1038/s41467-021-27070-5)
Supplement: Supplementary file 2 — Reporting Summary [file 41467_2021_27070_MOESM2_ESM.pdf]

nature research

Corresponding author(s): Jeffrey J. Tabor

Last updated by author(s): Oct 29, 2021

Reporting Summary

Nature Research wishes to improve the reproducibility of the work that we publish. This form provides structure for consistency and transparency in reporting. For further information on Nature Research policies, see our Editorial Policies and the Editorial Policy Checklist.

Statistics

For all statistical analyses, confirm that the following items are present in the figure legend, table legend, main text, or Methods section.

n/a	Confirmed	
☐	☒	The exact sample size (*n*) for each experimental group/condition, given as a discrete number and unit of measurement
☐	☒	A statement on whether measurements were taken from distinct samples or whether the same sample was measured repeatedly
☒	☐	The statistical test(s) used AND whether they are one- or two-sided<br>*Only common tests should be described solely by name; describe more complex techniques in the Methods section.*
☒	☐	A description of all covariates tested
☒	☐	A description of any assumptions or corrections, such as tests of normality and adjustment for multiple comparisons
☐	☒	A full description of the statistical parameters including central tendency (e.g. means) or other basic estimates (e.g. regression coefficient) AND variation (e.g. standard deviation) or associated estimates of uncertainty (e.g. confidence intervals)
☒	☐	For null hypothesis testing, the test statistic (e.g. *F*, *t*, *r*) with confidence intervals, effect sizes, degrees of freedom and *P* value noted<br>*Give P values as exact values whenever suitable.*
☒	☐	For Bayesian analysis, information on the choice of priors and Markov chain Monte Carlo settings
☒	☐	For hierarchical and complex designs, identification of the appropriate level for tests and full reporting of outcomes
☒	☐	Estimates of effect sizes (e.g. Cohen's *d*, Pearson's *r*), indicating how they were calculated

Our web collection on statistics for biologists contains articles on many of the points above.

Software and code

Policy information about availability of computer code

Data collection	FlowJo CE (v7.5.110.7) software was used to interface with the flow cytometer during data collection.
Data analysis	Flow cytometry processing and analysis: FlowCal (1.2.2), Python (3.7), custom Python code (https://github.com/taborlab/NoiseControl).<br>Fig. 2 inducer-mean transfer functions: R (3.6.3), R packages minpack.lm (1.2-1).<br>Fig. 3 mean-noise model fitting: Python 3.7, Python packages lmfit (1.0.0).<br>Dynamic area and dynamic noise: R (3.6.3), R packages alphahull (2.2) and sp (1.4-1), custom R code (https://github.com/taborlab/NoiseControl).<br>Fig. 4 convolution simulations, Bhattacharyya coeffecients, concordance: R (3.6.3).<br>Fig. 5 LOTUS model fitting: R (3.6.4), R packages minpack.lm (1.2-1).<br>Deterministic kinetic modeling: Custom Matlab (2019b) code (https://github.com/taborlab/NoiseControl)<br>Stochastic kinetic modeling: Matlab package SimBiology (2015a), custom Matlab code (https://github.com/taborlab/NoiseControl)

For manuscripts utilizing custom algorithms or software that are central to the research but not yet described in published literature, software must be made available to editors and reviewers. We strongly encourage code deposition in a community repository (e.g. GitHub). See the Nature Research guidelines for submitting code & software for further information.

Data

Policy information about availability of data

All manuscripts must include a data availability statement. This statement should provide the following information, where applicable:

- Accession codes, unique identifiers, or web links for publicly available datasets
- A list of figures that have associated raw data
- A description of any restrictions on data availability

Sequences of all plasmids used in this study are available from Genbank via the accession numbers listed in Supplementary Table 13. Flow cytometry datasets generated during this study have been deposited on GitHub (https://github.com/taborlab/NoiseControl). Any other relevant data can be obtained from the authors upon reasonable request.

Field-specific reporting

Please select the one below that is the best fit for your research. If you are not sure, read the appropriate sections before making your selection.

☒ Life sciences ☐ Behavioural & social sciences ☐ Ecological, evolutionary & environmental sciences

For a reference copy of the document with all sections, see nature.com/documents/nr-reporting-summary-flat.pdf

Life sciences study design

All studies must disclose on these points even when the disclosure is negative.

Sample size	Bacterial populations of 20,000 or more cells were measured by flow cytometry which yields about 6,000 events after gating. These gated and ungated sample sizes comport with standards in the field and provide a relative standard error of about 0.3-4% depending on individual sample CV (0.25 to 3 in this study).
Data exclusions	Cytometry data: An SSC threshold below a level characteristic of the bacterial populations was applied during acquisition such that events below the threshold (debris) were not detected. The first 250 events and final 100 events (fluidic transients), and events with values in the first or last bin (instrument detection limits) of the FSC, SSC, FL1, or FL3 channels were removed. A final density gate of 30% was applied to identify bacterial cell populations. This gating strategy was established prior to acquisition.<br>All other data: No data were excluded.
Replication	Experiments were repeated on one, two or three separate days as indicated in figure legends.
Randomization	Biological samples were not randomized as position in multi-well plates and cytometry acquisition order is not expected to affect measurements. Inducer concentrations were not randomized between wells (for the same reason as above) but were evenly sampled across the number of experiments performed. For example, inducer groups (by increasing concentration) 1, 3, 5, ..., etc. were sampled on day 1 and inducer groups 2,4,6,..., etc. were tested on day 2.
Blinding	Researchers were not blinded because (a) cells measurements were made by flow cytometry and therefore based on an unbiased sampling of cells within a well mixed culture, (b) data analysis was automated, (c) qualitative scoring metrics were not used in this study.

Reporting for specific materials, systems and methods

We require information from authors about some types of materials, experimental systems and methods used in many studies. Here, indicate whether each material, system or method listed is relevant to your study. If you are not sure if a list item applies to your research, read the appropriate section before selecting a response.

Materials & experimental systems

n/a	Involved in the study	
☒	☐	Antibodies
☒	☐	Eukaryotic cell lines
☒	☐	Palaeontology and archaeology
☒	☐	Animals and other organisms
☒	☐	Human research participants
☒	☐	Clinical data
☒	☐	Dual use research of concern

Methods

n/a	Involved in the study	
☒	☐	ChIP-seq
☐	☒	Flow cytometry
☒	☐	MRI-based neuroimaging

Flow Cytometry

Plots

Confirm that:

☒ The axis labels state the marker and fluorochrome used (e.g. CD4-FITC).

☒ The axis scales are clearly visible. Include numbers along axes only for bottom left plot of group (a 'group' is an analysis of identical markers).

☐ All plots are contour plots with outliers or pseudocolor plots.

☒ A numerical value for number of cells or percentage (with statistics) is provided.

Methodology

Sample preparation	Cell samples were transferred to an ice water bath for >= 15 min to arrest growth. 200 µL of each sample was transferred to a flow cytometry tube containing 1 mL phosphate buffered saline (PBS) for measurement.
Instrument	BD FACScan flow cytometer outfitted with blue (488 nm, 30 mW) and yellow (561 nm, 50 mW) solid-state lasers (Cytek). sfGFP fluorescence was measured in the FL1 channel with a 510/20 nm emission filter and mCherry fluorescence was measured in the FL3 channel with 650 nm long pass filter.
Software	Cytometer software: FlowJo CE (v7.5.110.7)<br>Cytometery processing and analysis: FlowCal (1.2.2), Python (3.7), custom Python code (https://github.com/taborlab/NoiseControl).
Cell population abundance	Event rates of 1,000–3,500 events/s were used, and all events were captured until 20,000 events occurred within an SSC-FSC area characteristic of the strain.
Gating strategy	An SSC threshold below a level characteristic of the bacterial populations was applied during acquisition such that events below the threshold (debris) were not detected. The first 250 events and final 100 events (fluidic transients), and events with values in the first or last bin (instrument detection limits) of the FSC, SSC, FL1, or FL3 channels were removed. A final density gate of 30% was applied to identify bacterial cell populations. This gating strategy was established prior to acquisition.

☒ Tick this box to confirm that a figure exemplifying the gating strategy is provided in the Supplementary Information.
